# Calibration: the Achilles heel of predictive analytics

**DOI:** 10.1186/s12916-019-1466-7

**Published:** 2019-12-16

**Authors:** Ben Van Calster, David J. McLernon, Maarten van Smeden, Laure Wynants, Ewout W. Steyerberg, On behalf of Topic Group ‘Evaluating diagnostic tests and prediction models’ of the STRATOS initiative, Patrick Bossuyt, Gary S. Collins, Petra Macaskill, David J. McLernon, Karel G. M. Moons, Ewout W. Steyerberg, Ben Van Calster, Maarten van Smeden, Andrew J. Vickers

**Affiliations:** 1 Department of Development and Regeneration, KU Leuven, Herestraat 49 box 805, 3000 Leuven, Belgium; 2 Department of Biomedical Data Sciences, Leiden University Medical Center, Leiden, Netherlands; 3 Medical Statistics Team, Institute of Applied Health Sciences, School of Medicine, Medical Sciences and Nutrition, University of Aberdeen, Aberdeen, UK; 4 Department of Clinical Epidemiology, Leiden University Medical Center, Leiden, Netherlands; 5 Department of Epidemiology, CAPHRI Care and Public Health Research Institute, Maastricht University, Maastricht, Netherlands; 6 http://www.stratos-initiative.org

**Keywords:** Calibration, Risk prediction models, Predictive analytics, Overfitting, Heterogeneity, Model performance

## Abstract

**Background:**

The assessment of calibration performance of risk prediction models based on regression or more flexible machine learning algorithms receives little attention.

**Main text:**

Herein, we argue that this needs to change immediately because poorly calibrated algorithms can be misleading and potentially harmful for clinical decision-making. We summarize how to avoid poor calibration at algorithm development and how to assess calibration at algorithm validation, emphasizing balance between model complexity and the available sample size. At external validation, calibration curves require sufficiently large samples. Algorithm updating should be considered for appropriate support of clinical practice.

**Conclusion:**

Efforts are required to avoid poor calibration when developing prediction models, to evaluate calibration when validating models, and to update models when indicated. The ultimate aim is to optimize the utility of predictive analytics for shared decision-making and patient counseling.

## Background

Medical predictive analytics have gained popularity in recent years, with numerous publications focusing on models that estimate patients’ risk of a disease or a future health state (the ‘event’) based on classical regression algorithms or modern flexible machine learning or artificial intelligence algorithms [1–3]. These predictions may support clinical decision-making and better inform patients. Algorithms (or risk prediction models) should give higher risk estimates for patients with the event than for patients without the event (‘discrimination’). Typically, discrimination is quantified using the area under the receiver operating characteristic curve (AUROC or AUC), also known as the concordance statistic or c-statistic. Additionally, it may be desirable to present classification performance at one or more risk thresholds such as sensitivity, specificity, and (stratum-specific) likelihood ratios. Herein, we focus on calibration, another key aspect of performance that is often overlooked. We define calibration, describe why it is important, outline causes for poor calibration, and summarize how calibration can be assessed.

## Main text

### Discrimination is important, but are the risk estimates reliable?

It is often overlooked that estimated risks can be unreliable even when the algorithms have good discrimination. For example, risk estimates may be systematically too high for all patients irrespective of whether they experienced the event or not. The accuracy of risk estimates, relating to the agreement between the estimated and observed number of events, is called ‘calibration’ [4]. Systematic reviews have found that calibration is assessed far less often than discrimination [2, 3, 5–7], which is problematic since poor calibration can make predictions misleading [8]. Previous work has highlighted that the use of different types of algorithms, varying from regression to flexible machine learning approaches, can lead to models that suffer greatly from poor calibration [9, 10]. Calibration has therefore been labeled the ‘Achilles heel’ of predictive analytics [11]. Reporting on calibration performance is recommended by the TRIPOD (Transparent Reporting of a multivariable prediction model for Individual Prognosis Or Diagnosis) guidelines for prediction modeling studies [12]. Calibration is especially important when the aim is to support decision-making, even when discrimination is moderate such as for breast cancer prediction models [13]. We explain the relevance of calibration in this paper and suggest solutions to prevent or correct poor calibration and thus make predictive algorithms more clinically relevant.

### How can inaccurate risk predictions be misleading?

If the algorithm is used to inform patients, poorly calibrated risk estimates lead to false expectations with patients and healthcare professionals. Patients may make personal decisions in anticipation of an event, or the absence thereof, that were in fact misguided. Take, for example, a prediction model that predicts the chance that in vitro fertilization (IVF) treatment leads to a live birth [14]. Irrespective of how well the models can discriminate between treatments that end in live birth versus those that do not, it is clear that strong over- or underestimation of the chance of a live birth makes the algorithms clinically unacceptable. For instance, a strong overestimation of the chance of live birth after IVF would give false hope to couples going through an already stressful and emotional experience. Treating a couple who, in reality, has a favorable prognosis exposes the woman unnecessarily to possible harmful side effects, e.g., ovarian hyperstimulation syndrome.

In fact, poor calibration may make an algorithm less clinically useful than a competitor algorithm that has a lower AUC but is well calibrated [8]. As an example, consider the QRISK2–2011 and NICE Framingham models to predict the 10-year risk of cardiovascular disease. An external validation study of these models in 2 million patients from the United Kingdom indicated that QRISK2–2011 was well calibrated and had an AUC of 0.771, whereas NICE Framingham was overestimating risk, with an AUC of 0.776 [15]. When using the traditional risk threshold of 20% to identify high-risk patients for intervention, QRISK2–2011 would select 110 per 1000 men aged between 35 and 74 years. On the other hand, NICE Framingham would select almost twice as many (206 per 1000 men) because a predicted risk of 20% based on this model actually corresponded to a lower event rate. This example illustrates that overestimation of risk leads to overtreatment. Conversely, underestimation leads to undertreatment.

### Why may an algorithm give poorly calibrated risk predictions?

Many possible sources may distort the calibration of risk predictions. A first set of causes relates to variables and characteristics unrelated to algorithm development. Often, patient characteristics and disease incidence or prevalence rates vary greatly between health centers, regions, and countries [16]. When an algorithm is developed in a setting with a high disease incidence, it may systematically give overestimated risk estimates when used in a setting where the incidence is lower [17]. For example, university hospitals may treat more patients with the event of interest than regional hospitals; such heterogeneity between settings can affect risk estimates and their calibration [18]. The predictors in the algorithm may explain a part of the heterogeneity, but often differences between predictors will not explain all differences between settings [19]. Patient populations also tend to change over time, e.g., due to changes in referral patterns, healthcare policy, or treatment policies [20, 21]. For example, in the last 10 years, there has been a drive in Europe to lower the number of embryos transferred in IVF and improvements in IVF cryopreservation technology led to an increase in embryo freezing and storage for subsequent transfer [22]; such evolutions may change the calibration of algorithms that predict IVF success [23].

A second set of causes relates to methodological problems regarding the algorithm itself. Statistical overfitting is common. It is caused by a modeling strategy that is too complex for the amount of data at hand (e.g., too many candidate predictors, predictor selection based on statistical significance, use of a very flexible algorithm such as a neural network) [24]. Overfitted predictions capture too much random noise in the development data. Thus, when validated on new data, an overfitted algorithm is expected to show lower discrimination performance and predicted risks that are too extreme – patients at high risk of the event tend to get overestimated risk predictions, whereas patients at low risk of the event tend to get underestimated risk predictions. Apart from statistical overfitting, medical data usually contain measurement error, for example, biomarker expressions vary with assay kits and ultrasound measurement of tumor vascularity has inter- and intra-observer variability [25, 26]. If measurement error systematically differs between settings (e.g., measurements of a predictor are systemically more biased upward in a different setting), this affects the predicted risks and thus calibration of an algorithm [27].

### How to assess calibration?

The concepts explained in this section are illustrated in Additional file 1, with the validation of the Risk of Ovarian Malignancy Algorithm (ROMA) for the diagnosis of ovarian malignancy in women with an ovarian tumor selected for surgical removal [28]; further details can be found elsewhere [1, 4, 29].

According to four increasingly stringent levels of calibration, models can be calibrated in the mean, weak, moderate, or strong sense [4]. First, to assess ‘mean calibration’ (or ‘calibration-in-the-large’), the average predicted risk is compared with the overall event rate. When the average predicted risk is higher than the overall event rate, the algorithm overestimates risk in general. Conversely, underestimation occurs when the observed event rate is higher than the average predicted risk.

Second, ‘weak calibration’ means that, on average, the model does not over- or underestimate risk and does not give overly extreme (too close to 0 and 1) or modest (too close to disease prevalence or incidence) risk estimates. Weak calibration can be assessed by the calibration intercept and calibration slope. The calibration slope evaluates the spread of the estimated risks and has a target value of 1. A slope < 1 suggests that estimated risks are too extreme, i.e., too high for patients who are at high risk and too low for patients who are at low risk. A slope > 1 suggests the opposite, i.e., that risk estimates are too moderate. The calibration intercept, which is an assessment of calibration-in-the-large, has a target value of 0; negative values suggest overestimation, whereas positive values suggest underestimation.

**Fig. 1:**
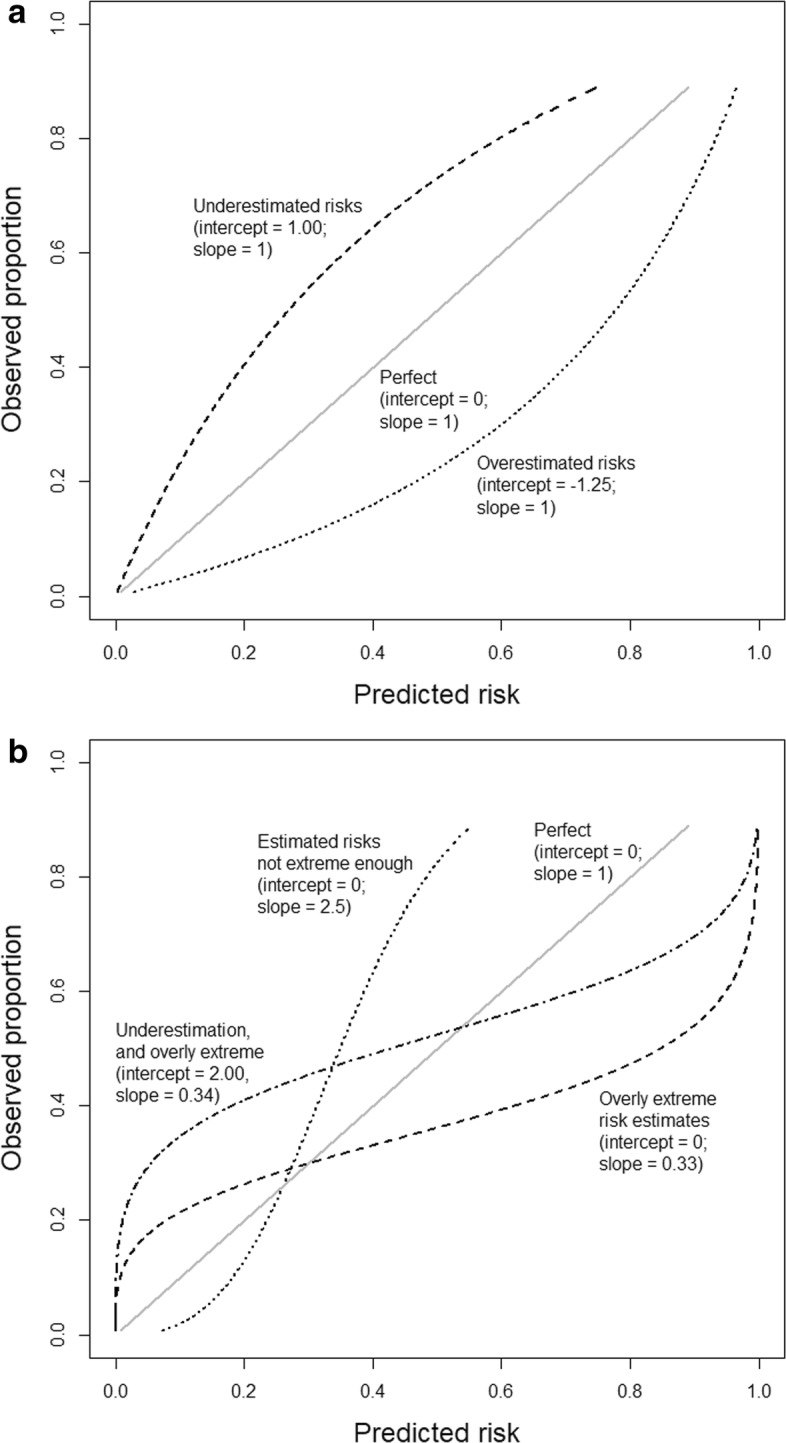
Illustrations of different types of miscalibration. Illustrations are based on an outcome with a 25% event rate and a model with an area under the ROC curve (AUC or c-statistic) of 0.71. Calibration intercept and slope are indicated for each illustrative curve. **a** General over- or underestimation of predicted risks. **b** Predicted risks that are too extreme or not extreme enough

Third, moderate calibration implies that estimated risks correspond to observed proportions, e.g., among patients with an estimated risk of 10%, 10 in 100 have or develop the event. This is assessed with a flexible calibration curve to show the relation between the estimated risk (on the x-axis) and the observed proportion of events (y-axis), for example, using loess or spline functions. A curve close to the diagonal indicates that predicted risks correspond well to observed proportions. We show a few theoretical curves in Fig. 1a,b, each of which corresponds to different calibration intercepts and slopes. Note that a calibration intercept close to 0 and a calibration slope close to 1 do not guarantee that the flexible calibration curve is close to the diagonal (see Additional file 1 for an example). To obtain a precise calibration curve, a sufficiently large sample size is required; a minimum of 200 patients with and 200 patients without the event has been suggested [4], although further research is needed to investigate how factors such as disease prevalence or incidence affect the required sample size [12]. In small datasets, it is defendable to evaluate only weak calibration by calculating the calibration intercept and slope.

Fourth, strong calibration means that the predicted risk corresponds to the observed proportion for every possible combination of predictor values; this implies that calibration is perfect and is a utopic goal [4].

The commonly used Hosmer–Lemeshow test is often presented as a calibration test, though it has many drawbacks – it is based on artificially grouping patients into risk strata, gives a *P* value that is uninformative with respect to the type and extent of miscalibration, and suffers from low statistical power [1, 4]. Therefore, we recommend against using the Hosmer–Lemeshow test to assess calibration.

### How to prevent or correct poor calibration?

When developing a predictive algorithm, the first step involves the control of statistical overfitting. It is important to prespecify the modeling strategy and to ensure that sample size is sufficient for the number of considered predictors [30, 31]. In smaller datasets, procedures that aim to prevent overfitting should be considered, e.g., using penalized regression techniques such as Ridge or Lasso regression [32] or using simpler models. Simpler models can refer to fewer predictors, omitting nonlinear or interaction terms, or using a less flexible algorithm (e.g., logistic regression instead of random forests or a priori limiting the number of hidden neurons in a neural network). However, using models that are too simple can backfire (Additional file 1), and penalization does not offer a miracle solution for uncertainty in small datasets [33]. Therefore, in small datasets, it is reasonable for a model not to be developed at all. Additionally, internal validation procedures can quantify the calibration slope. At internal validation, calibration-in-the-large is irrelevant since the average of predicted risks will match the event rate. In contrast, calibration-in-the-large is highly relevant at external validation, where we often note a mismatch between the predicted and observed risks.

When we find poorly calibrated predictions at validation, algorithm updating should be considered to provide more accurate predictions for new patients from the validation setting [1, 20]. Updating of regression-based algorithms may start with changing the intercept to correct calibration-in-the-large [34]. Full refitting of the algorithm, as in the case study below, will improve calibration if the validation sample is relatively large [35]. We present a detailed illustration of updating of the ROMA model in Additional file 1. Continuous updating strategies are also gaining in popularity; such strategies dynamically address shifts in the target population over time [36].

### Published case study on the diagnosis of obstructive coronary artery disease

**Fig. 2:**
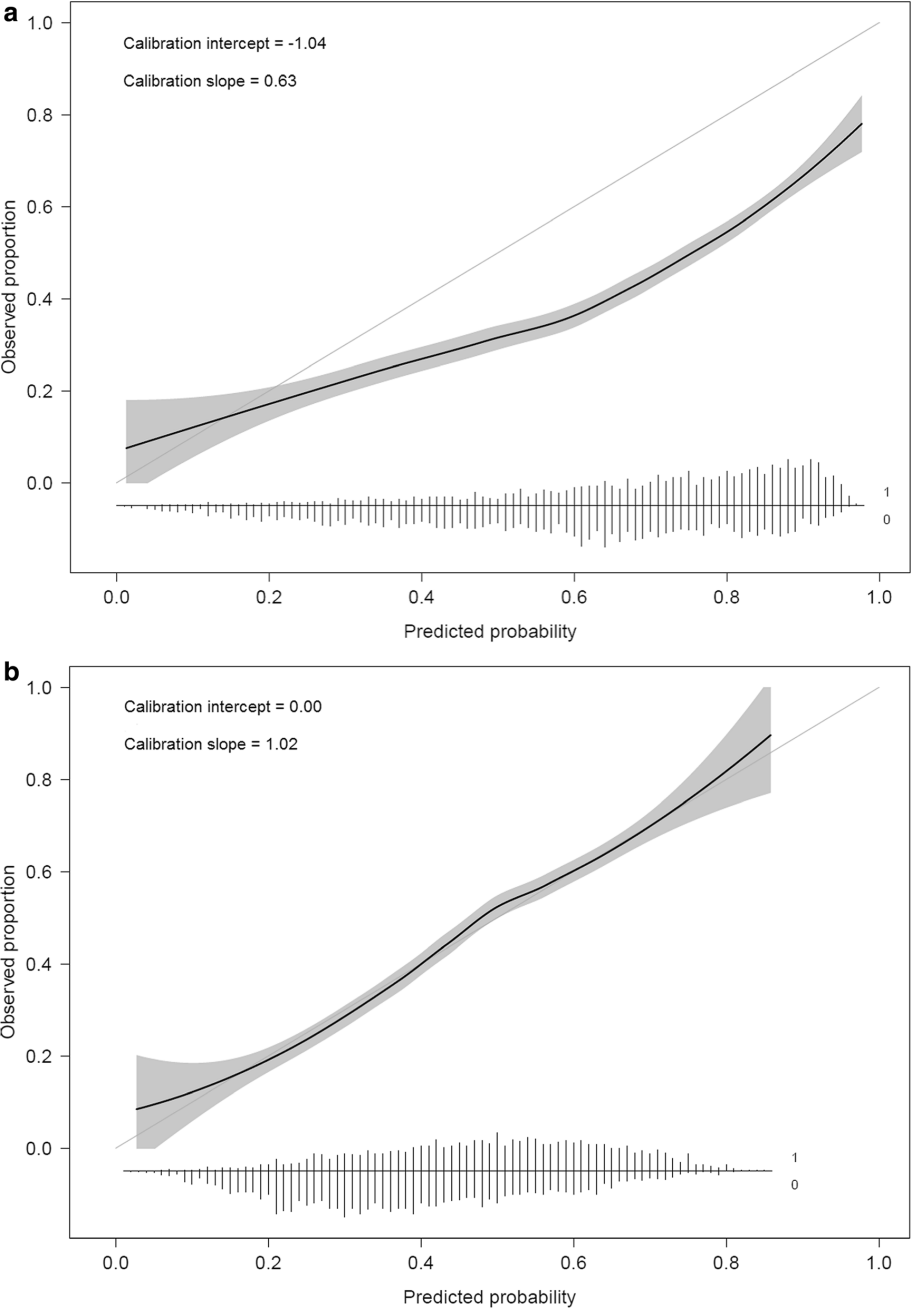
Calibration curves when validating a model for obstructive coronary artery disease before and after updating. **a** Calibration curve before updating. **b** Calibration curve after updating by re-estimating the model coefficients. The flexible curve with pointwise confidence intervals (gray area) was based on local regression (loess). At the bottom of the graphs, histograms of the predicted risks are shown for patients with (1) and patients without (0) coronary artery disease. Figure adapted from Edlinger et al. [38], which was published under the Creative Commons Attribution–Noncommercial (CC BY-NC 4.0) license

Consider a logistic regression model to predict obstructive coronary artery disease (oCAD) in patients with stable chest pain and without a medical history of oCAD [37]. The model was developed on data from 5677 patients recruited at 18 European and American centers, of whom 31% had oCAD. The algorithm was externally validated on data from 4888 patients in Innsbruck, Austria, of whom 44% had oCAD [38]. The algorithm had an AUC of 0.69. Calibration suggested a combination of overestimated (intercept − 1.04) and overly extreme risk predictions (slope 0.63) (Fig. 2a). Calibration was improved by refitting the model, i.e., by re-estimating the predictor coefficients (Fig. 2b).

## Conclusions

The key arguments of this paper are summarized in Table 1. Poorly calibrated predictive algorithms can be misleading, which may result in incorrect and potentially harmful clinical decisions. Therefore, we need prespecified modeling strategies that are reasonable with respect to the available sample size. When validating algorithms it is imperative to evaluate calibration using appropriate measures and visualizations – this helps us to understand how the algorithm performs in a particular setting, where predictions may go wrong, and whether the algorithm can benefit from updating. Due to local healthcare systems and referral patterns, population differences between centers and regions are expected; it is likely that prediction models do not include all the predictors needed to accommodate these differences. Together with the phenomenon of population drifts, models ideally require continued monitoring in local settings in order to maximize their benefit over time. This argument will become even more vital with the growing popularity of highly flexible algorithms. The ultimate aim is to optimize the utility of predictive analytics for shared decision-making and patient counseling.

**Table 1:** Summary points on calibration

Why calibration matters	- Decisions are often based on risk, so predicted risks should be reliable
	- Poor calibration may make a prediction model clinically useless or even harmful
Causes of poor calibration	- Statistical overfitting and measurement error
	- Heterogeneity in populations in terms of patient characteristics, disease incidence or prevalence, patient management, and treatment policies
Assessment of calibration in practice	- Perfect calibration, where predicted risks are correct for every covariate pattern, is utopic; we should not aim for that
	- At model development, focus on nonlinear effects and interaction terms only if a sufficiently large sample size is available; low sample sizes require simpler modeling strategies or that no model is developed at all
	- Avoid the Hosmer–Lemeshow test to assess or prove calibration
	- At internal validation, focus on the calibration slope as a part of the assessment of statistical overfitting
	- At external validation, focus on the calibration curve, intercept and slope
	- Model updating should be considered in case of poor calibration; re-estimating the model entirely requires sufficient data

## Supplementary information

**Additional file 1.** Detailed illustration of the assessment of calibration and model updating: the ROMA logistic regression model.

## Data Availability

This study did not use data. Figure 2 was adapted from Edlinger et al. [38], which was published under the Creative Commons Attribution Non Commercial (CC BY-NC 4.0) license.

## References

[1] Steyerberg EW (2009). Clinical prediction models.

[2] Wessler BS, Paulus J, Lundquist CM (2017). Tufts PACE clinical predictive model registry: update 1990 through 2015. Diagn Progn Res.

[3] Kleinrouweler CE, Cheong-See FM, Collins GS (2016). Prognostic models in obstetrics: available, but far from applicable. Am J Obstet Gynecol.

[4] Van Calster B, Nieboer D, Vergouwe Y, De Cock B, Pencina MJ, Steyerberg EW (2016). A calibration hierarchy for risk models was defined: from utopia to empirical data. J Clin Epidemiol.

[5] Collins GS, de Groot JA, Dutton S (2014). External validation of multivariable prediction models: a systematic review of methodological conduct and reporting. BMC Med Res Methodol.

[6] Christodoulou E, Ma J, Collins GS, Steyerberg EW, Verbakel JY, Van Calster B (2019). A systematic review shows no performance benefit of machine learning over logistic regression for clinical prediction models. J Clin Epidemiol.

[7] Bouwmeester W, Zuithoff NPA, Mallett S (2012). Reporting and methods in clinical prediction research: a systematic review. PLoS Med.

[8] Van Calster B, Vickers AJ (2015). Calibration of risk prediction models: impact on decision-analytic performance. Med Decis Mak.

[9] Van Hoorde K, Van Huffel S, Timmerman D, Bourne T, Van Calster B (2015). A spline-based tool to assess and visualize the calibration of multiclass risk predictions. J Biomed Inform.

[10] Van der Ploeg T, Nieboer D, Steyerberg EW (2016). Modern modeling techniques had limited external validity in predicting mortality from traumatic brain injury. J Clin Epidemiol.

[11] Shah ND, Steyerberg EW, Kent DM (2018). Big data and predictive analytics: recalibrating expectations. JAMA..

[12] Moons KG, Altman DG, Reitsma JB (2015). Transparent Reporting of a multivariable prediction model for Individual Prognosis or Diagnosis (TRIPOD): explanation and elaboration. Ann Intern Med.

[13] Yala A, Lehman C, Schuster T, Portnoi T, Barzilay R (2019). A deep learning mammography-based model for improved breast cancer risk prediction. Radiology..

[14] Dhillon RK, McLernon DJ, Smith PP (2016). Predicting the chance of live birth for women undergoing IVF: a novel pretreatment counselling tool. Hum Reprod.

[15] Collins GS, Altman DG (2012). Predicting the 10 year risk of cardiovascular disease in the United Kingdom: independent and external validation of an updated version of QRISK2. BMJ..

[16] Bray F, Ferlay J, Soerjomataram I, Siegel RL, Torre LA, Jemal A (2018). Global cancer statistics 2018: GLOBOCAN estimates of incidence and mortality worldwide for 36 cancers in 185 countries. CA Cancer J Clin.

[17] Testa A, Kaijser J, Wynants L (2014). Strategies to diagnose ovarian cancer: new evidence from phase 3 of the multicentre international IOTA study. Br J Cancer.

[18] Riley RD, Ensor J, Snell KI (2016). External validation of clinical prediction models using big datasets from e-health records or IPD meta-analysis: opportunities and challenges. BMJ..

[19] Steyerberg EW, Roobol MJ, Kattan MW, van der Kwast TH, de Koning HJ, Schröder FH (2007). Prediction of indolent prostate cancer: validation and updating of a prognostic nomogram. J Urol.

[20] Davis SE, Lasko TA, Chen G, Siew ED, Matheny ME (2017). Calibration drift in regression and machine learning models for acute kidney injury. J Am Med Inform Assoc.

[21] Thai TN, Ebell MH (2019). Prospective validation of the good outcome following attempted resuscitation (GO-FAR) score for in-hospital cardiac arrest prognosis. Resuscitation..

[22] Leijdekkers JA, Eijkemans MJC, van Tilborg TC (2018). Predicting the cumulative chance of live birth over multiple complete cycles of in vitro fertilization: an external validation study. Hum Reprod.

[23] te Velde ER, Nieboer D, Lintsen AM (2014). Comparison of two models predicting IVF success; the effect of time trends on model performance. Hum Reprod.

[24] Steyerberg EW, Uno H, Ioannidis JPA, Van Calster B (2018). Poor performance of clinical prediction models: the harm of commonly applied methods. J Clin Epidemiol.

[25] Murthy V, Rishi A, Gupta S (2016). Clinical impact of prostate specific antigen (PSA) inter-assay variability on management of prostate cancer. Clin Biochem.

[26] Wynants L, Timmerman D, Bourne T, Van Huffel S, Van Calster B (2013). Screening for data clustering in multicenter studies: the residual intraclass correlation. BMC Med Res Methodol.

[27] Luijken K, Groenwold RHH, Van Calster B, Steyerberg EW, van Smeden M (2019). Impact of predictor measurement heterogeneity across settings on performance of prediction models: a measurement error perspective. Stat Med.

[28] Moore RG, McMeekin DS, Brown AK (2009). A novel multiple marker bioassay utilizing HE4 and CA125 for the prediction of ovarian cancer in patients with a pelvic mass. Gynecol Oncol.

[29] Austin PC, Steyerberg EW (2014). Graphical assessment of internal and external calibration of logistic regression models by using loess smoothers. Stat Med.

[30] van Smeden M, Moons KGM, de Groot JA (2019). Sample size for binary logistic prediction models: beyond events per variable criteria. Stat Meth Med Res..

[31] Riley RD, Snell KIE, Ensor J (2019). Minimum sample size for developing a multivariable prediction model: PART II - binary and time-to-event outcomes. Stat Med.

[32] Moons KGM, Donders AR, Steyerberg EW, Harrell FE (2004). Penalized maximum likelihood estimation to directly adjust diagnostic and prognostic prediction models for overoptimism: a clinical example. J Clin Epidemiol.

[33] Van Calster B, van Smeden M, Steyerberg EW. On the variability of regression shrinkage methods for clinical prediction models: simulation study on predictive performance. arXiv. 2019; https://arxiv.org/abs/1907.11493. Accessed 10 Oct 2019.

[34] Steyerberg EW, Borsboom GJJM, van Houwelingen HC, Eijkemans MJC, Habbema JDF (2004). Validation and updating of predictive logistic regression models: a study on sample size and shrinkage. Stat Med.

[35] Su TL, Jaki T, Hickey GL, Buchan I, Sperrin M (2018). A review of statistical updating methods for clinical prediction models. Stat Meth Med Res.

[36] Hickey GL, Grant SW, Caiado C (2013). Dynamic prediction modeling approaches for cardiac surgery. Circ Cardiovasc Qual Outcomes.

[37] Genders TSS, Steyerberg EW, Hunink MG (2012). Prediction model to estimate presence of coronary artery disease: retrospective pooled analysis of existing cohorts. BMJ..

[38] Edlinger M, Wanitschek M, Dörler J, Ulmer H, Alber HF, Steyerberg EW (2017). External validation and extension of a diagnostic model for obstructive coronary artery disease: a cross-sectional predictive evaluation in 4888 patients of the Austrian Coronary Artery disease Risk Determination In Innsbruck by diaGnostic ANgiography (CARDIIGAN) cohort. BMJ Open.

