# Supplementary material for: Calibration: the Achilles heel of predictive analytics
Source: BMC Med. 2019 Dec 16;17:230. doi: 10.1186/s12916-019-1466-7 (PMC6912996; doi:10.1186/s12916-019-1466-7)

**Calibration: the Achilles heel of predictive analytics**

**Additional file 1.** Detailed illustration of the assessment of calibration and model updating: the ROMA logistic regression model

1. **Introduction**

The Risk of Ovarian Malignancy Algorithm (ROMA) model was developed to predict the risk of epithelial ovarian cancer in women with an ovarian tumor selected for surgery [1]. The model uses three predictors: menopausal status (post, coded as 0 for premenopausal and 1 for menopausal), human epididymis secretory protein 4 (HE4), and tumor marker cancer antigen 125 (CA125). The authors give separate formulas for pre- and postmenopausal patients, which means that ROMA includes interaction terms between post and HE4 and between post and CA125. The ROMA model is therefore given as:

LP_ROMA_ = –12.0 + 3.91*post + 2.38*log(HE4) + 0.0626*log(CA125) – 1.34*post*log(HE4) + 0.6694*post*log(CA125)

where log() means the natural log. The risk of malignancy is then obtained as follows:

R_ROMA_ = exp(LP_ROMA_) / (1 + exp(LP_ROMA_))

We will first evaluate the external predictive performance of the ROMA model on data from 360 patients recruited at the University Hospitals Leuven, Belgium, between 2005 and 2009 [2]. We will follow common practice by evaluating the performance of ROMA to predict the risk of any type ovarian malignance (instead of epithelial ovarian cancer only). This means that the outcome in our evaluation is changed from malignant epithelial ovarian cancer (as in model development) to any ovarian malignancy (event; includes borderline malignant tumors, non-epithelial ovarian cancer, and secondary metastatic cancer) versus a benign tumor (non-event). Although epithelial ovarian cancer is the most common type of ovarian malignancy, this change in the definition of the outcome is likely to induce some miscalibration by underestimating the risk of ovarian malignancy.

As a second step we will illustrate updating the ROMA model.

1. **Validation**
   1. Discrimination

The area under the ROC curve (AUC) was estimated at 0.89 (95% CI 0.85–0.92), which suggests very good discrimination between benign and malignant ovarian tumors of the ROMA model in our data.

- 1. Mean calibration (calibration-in-the-large)

The prevalence of malignancy was 40% (144/360). The average estimated risk given by ROMA was 0.308 (i.e., 30.8%), which indicates that there is a tendency to give underestimated risks by the ROMA model.

- 1. Weak calibration (calibration intercept and calibration slope)

To estimate the calibration intercept, we fit the logistic model Y = α + LP_ROMA_. In logistic regression, Y is the logarithm of the estimated risk (R_ROMA_) divided by 1 minus the estimated risk, i.e., log(R_ROMA_/(1–R_ROMA_)). Notice that there is no regression coefficient for the effect of LP_ROMA_, which is equivalent to setting the coefficient of LP_ROMA_ to 1. In a practical sense, this means that a regression model is fitted with LP_ROMA_ as an ‘offset term’. The estimated value of the intercept α is the calibration intercept.

To obtain the calibration slope, we fit the logistic model Y = α’ + ß*LP_ROMA_. The estimated value of the slope ß is the calibration slope.

In our dataset, the calibration intercept of ROMA was 0.87 (95% CI 0.57–1.16), with zero as the target value, suggesting the tendency to give underestimated risks. The calibration slope was 1.01 (95% CI 0.79–1.23), which is very close to the target value of 1. This calibration slope suggests that risk estimates were not systematically too extreme or moderate.

- 1. Moderate calibration (flexible calibration curve)

The flexible calibration curve is based on the logistic model Y = α” + *f*(LP_ROMA_). F is a flexible smoothed continuous function based on loess or spline functions, for example. In our example, we used a loess curve. This curve showed a strong level of underestimation of risks across the range of true risks (Fig. S1). We generated the calibration curves using the val.prob.ci.2 function from the CalibrationCurves package for R (<https://github.com/BavoDC/CalibrationCurves>). R version 3.5.3 was used ([www.R-project.org](http://www.R-project.org)).

- 1. Hosmer–Lemeshow test

The Hosmer–Lemeshow test, using the standard number of 10 groups, has a *P* <0.0001. This suggests poor calibration but does not give any further useful information in terms of degree and type of miscalibration. Additionally, as always, the *P* value depends on the sample size. We advise against the use of this test (see main paper). Figure S2 presents a grouped calibration plot, showing the data behind the Hosmer–Lemeshow test. For each of the 10 groups (based on deciles of estimated risk), the average estimated risk (x-axis) is plotted against the observed proportion of patients with a malignancy (y-axis).

1. **Updating of a logistic regression model**
   1. Updating using intercept adjustment

Intercept adjustment is the simplest form of model updating. Of the model’s original regression coefficients, only the intercept is re-estimated. Updating can be considered when the calibration intercept is not close to 0, as in our example. Adjusting the intercept is done by adding the calibration intercept (0.87) to the model intercept (–12.0). The new, updated LP of the ROMA model then becomes:

LP_ROMA_IA_ = –11.13 + 3.91*post + 2.38*log(HE4) + 0.0626*log(CA125) – 1.34*post*log(HE4) + 0.6694*post*log(CA125)

After this intercept update, the flexible calibration curve of the intercept-adjusted model is close to the diagonal reference line of perfect moderate calibration (Fig. S3).

- 1. Updating using intercept and slope adjustment

This adjustment is slightly more elaborate than simple intercept adjustment. Apart from estimating a new intercept, we also update the coefficients of the individual predictors by multiplying each predictor with a factor that is the estimated calibration slope. In our example, we can multiply all model coefficients with the calibration slope (1.014), and add the estimate of α’ (0.89; the intercept of the calibration slope model). The new LP then becomes:

LP_ROMA_ISA_ = –11.11 + 3.965*post + 2.413*log(HE4) + 0.06348*log(CA125) – 1.359*post*log(HE4) + 0.6788*post*log(CA125)

The flexible calibration curve of the intercept and slope-adjusted model is similar to the curve for the intercept-adjusted model (Fig. S4). This is as expected, because the estimated calibration slope of the ROMA model was very close to the target value of 1.

- 1. Updating using re-estimation of the full model (refit)

This adjustment is again more elaborate. We now determine new values for the model intercept and for every coefficient in the model. In essence, a new prediction model is developed, which requires a sufficiently large sample size. When the sample size is small and/or the number of coefficients is large, this method has the tendency to overfit the development data.

The re-estimated ROMA model in our data is as follows:

LP_ROMA_refit_ = –10.84 + 5.41*post + 1.65*log(HE4) + 0.753*log(CA125) – 1.22*post*log(HE4) + 0.261*post*log(CA125)

The flexible calibration curve of the re-estimated model is similar to the previous curves (Fig. S5), which were already close to the diagonal. Notice that re-estimation of all coefficients can also change the AUC. This model has an AUC of 0.91 (vs. 0.89 for the original ROMA model).

Of course, the results of updated versions of the model require additional testing in independent data.

1. **The impact of nonlinear and interaction terms**

When developing a prediction model using standard logistic regression, the calibration intercept and slope are, by definition, 0 and 1 on the development data. However, poor modeling of nonlinear and interaction terms may still result in a poor flexible calibration curve. In such situations, weak calibration is perfect but moderate calibration can be poor even on the development data. Experience suggests that poor modeling of nonlinear effects of predictors is more important for calibration than poor modeling of interaction effects. Therefore, if data allow, nonlinear effects of continuous predictors should be considered. With respect to modeling of interaction terms, we advise more care. The number of possible interaction effects grows exponentially with the number of predictors; therefore, when a large number of predictors is considered, very large datasets are needed to reliably select and model interaction terms. Here, common advice is to limit the analysis to interaction terms that are likely to be important a priori, if any.

If we refit the ROMA model without the interaction terms, we get the following:

LP_ROMA_refit_main_ = –8.24 + 1.28*post + 0.904*log(HE4) + 0.859*log(CA125).

The flexible calibration curve of this re-estimated model, including only the main effects, is nearly identical to the curve for the fully re-estimated model (Fig. S6).

If we refit the ROMA model without the natural log transformation (i.e., with only linear effects on the original scale) and interaction terms, we get the following:

LP_ROMA_refit_mainlin_ = –2.55 + 1.37*post + 0.00541*HE4 + 0.00671*CA125.

The flexible calibration curve of this re-estimated model, including only the linear main effect, shows that the calibration is poor (Fig. S7). Thus, this re-estimation approach suggests that nonlinear effects of HE4 and CA125 lead to a better fit than linear effects in the updating data.

1. **References**
2. Moore RG, McMeekin DS, Brown AK, et al. A novel multiple marker bioassay utilizing HE4 and CA125 for the prediction of ovarian cancer in patients with a pelvic mass. Gynecol Oncol. 2009;112:40–6.
3. Kaijser J, Van Gorp T, Van Hoorde K, et al. A comparison between an ultrasound based prediction model (LR2) and the risk of ovarian malignancy algorithm (ROMA) to assess the risk of malignancy in women with an adnexal mass. Gynecol Oncol. 2013;129:377–83.

Fig. S1 Flexible calibration curve of the original ROMA model.


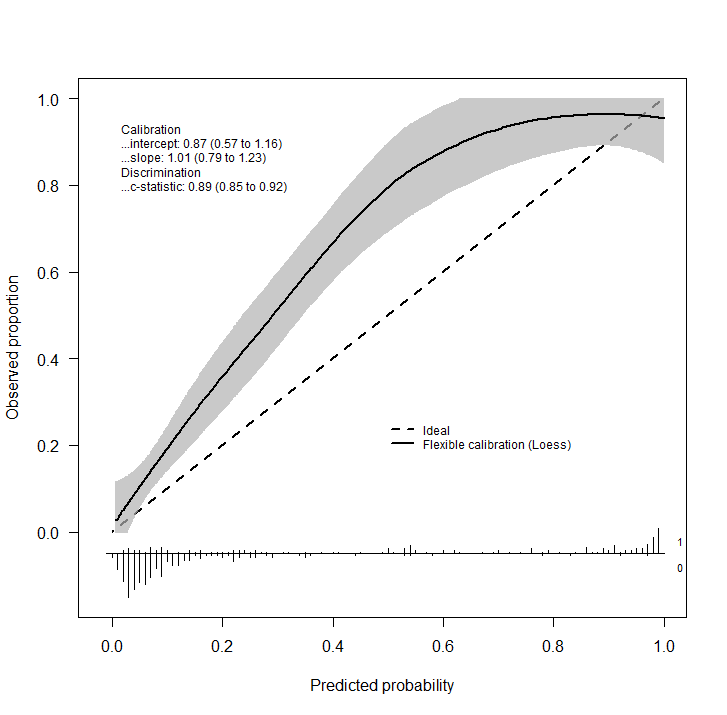


Fig. S2. Grouped calibration plot of the original ROMA model using 10 groups.


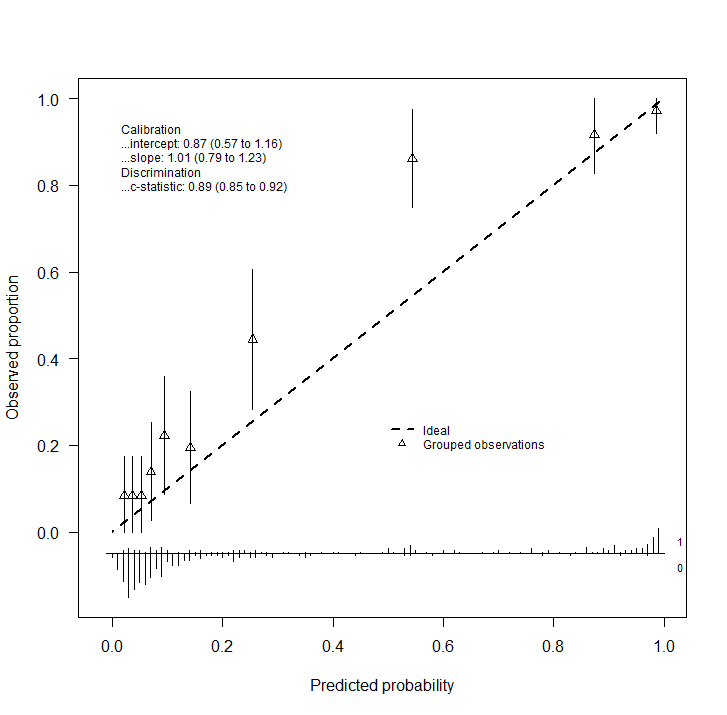


Fig. S3 Flexible calibration curve of the intercept-adjusted ROMA model.


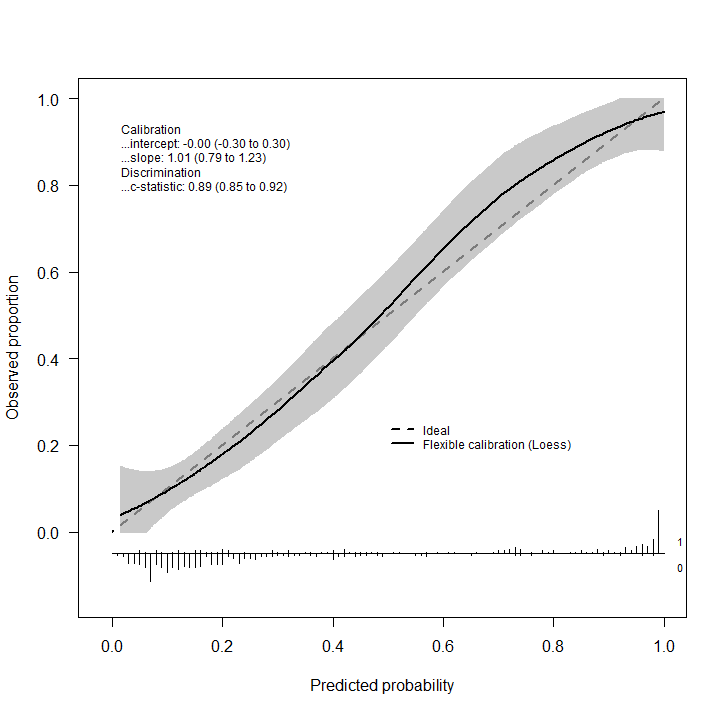


Fig. S4 Flexible calibration curve of the intercept and slope-adjusted ROMA model.


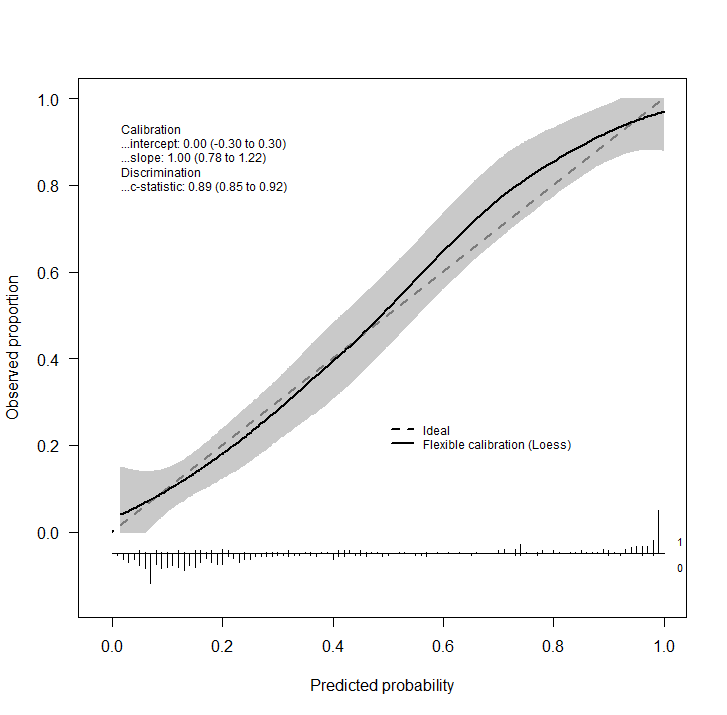


Fig. S5 Flexible calibration curve of the re-estimated ROMA model.


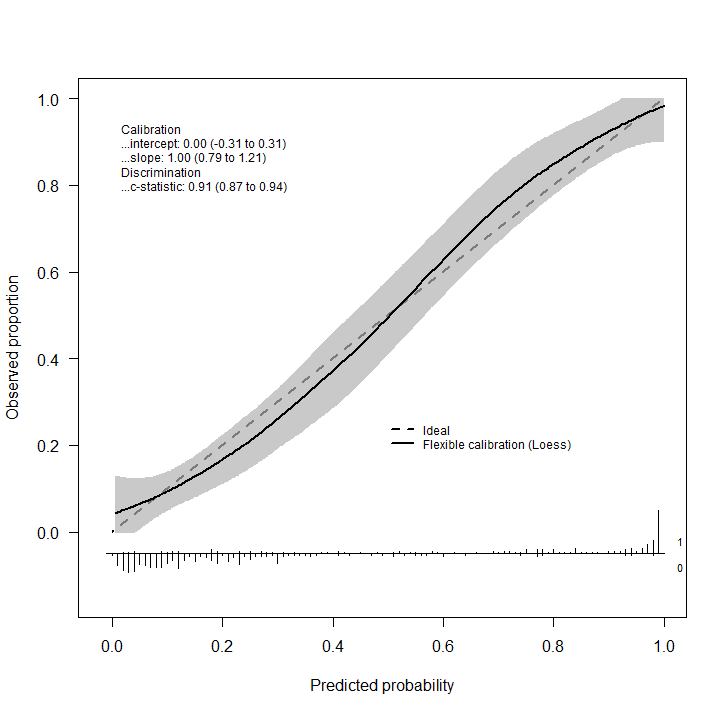


Fig. 6 Flexible calibration curve of the re-estimated ROMA model without interaction terms.


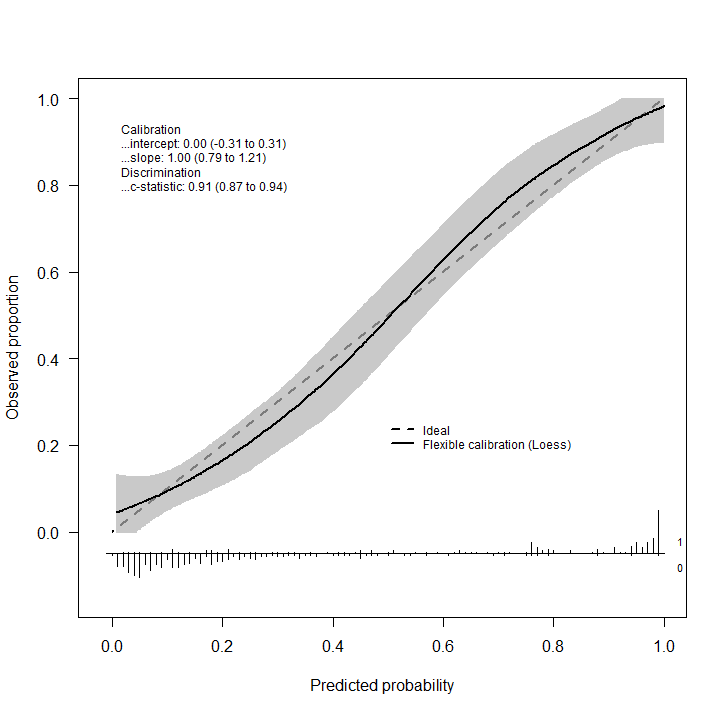


Fig. S7 Flexible calibration curve of the re-estimated ROMA model

without interaction or nonlinear terms.


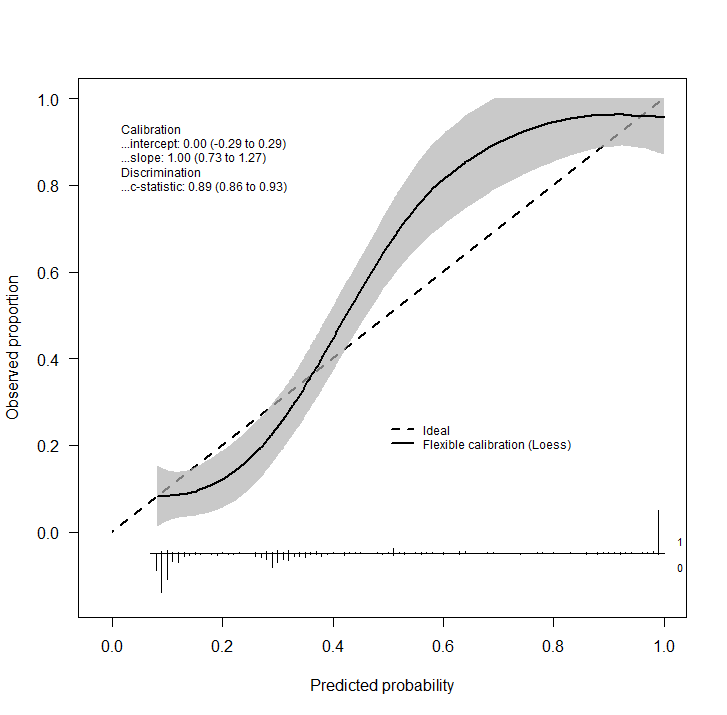

Supplement: Supplementary file 1 — Additional file 1. Detailed illustration of the assessment of calibration and model updating: the ROMA logistic regression model. [file 12916_2019_1466_MOESM1_ESM.docx]
